# First person – Astrid M. Baattrup

**DOI:** 10.1242/dmm.052698

**Published:** 2025-12-12

## Abstract

First Person is a series of interviews with the first authors of a selection of papers published in Disease Models & Mechanisms, helping researchers promote themselves alongside their papers. Astrid M. Baattrup is first author on ‘ ETV1 is a key regulator of enteroendocrine PYY production’, published in DMM. Astrid is a postdoc in the lab of Kim B. Jensen at University of Copenhagen, Copenhagen, Denmark, investigating how enteroendocrine cells in the gut are regulated transcriptionally and functionally.


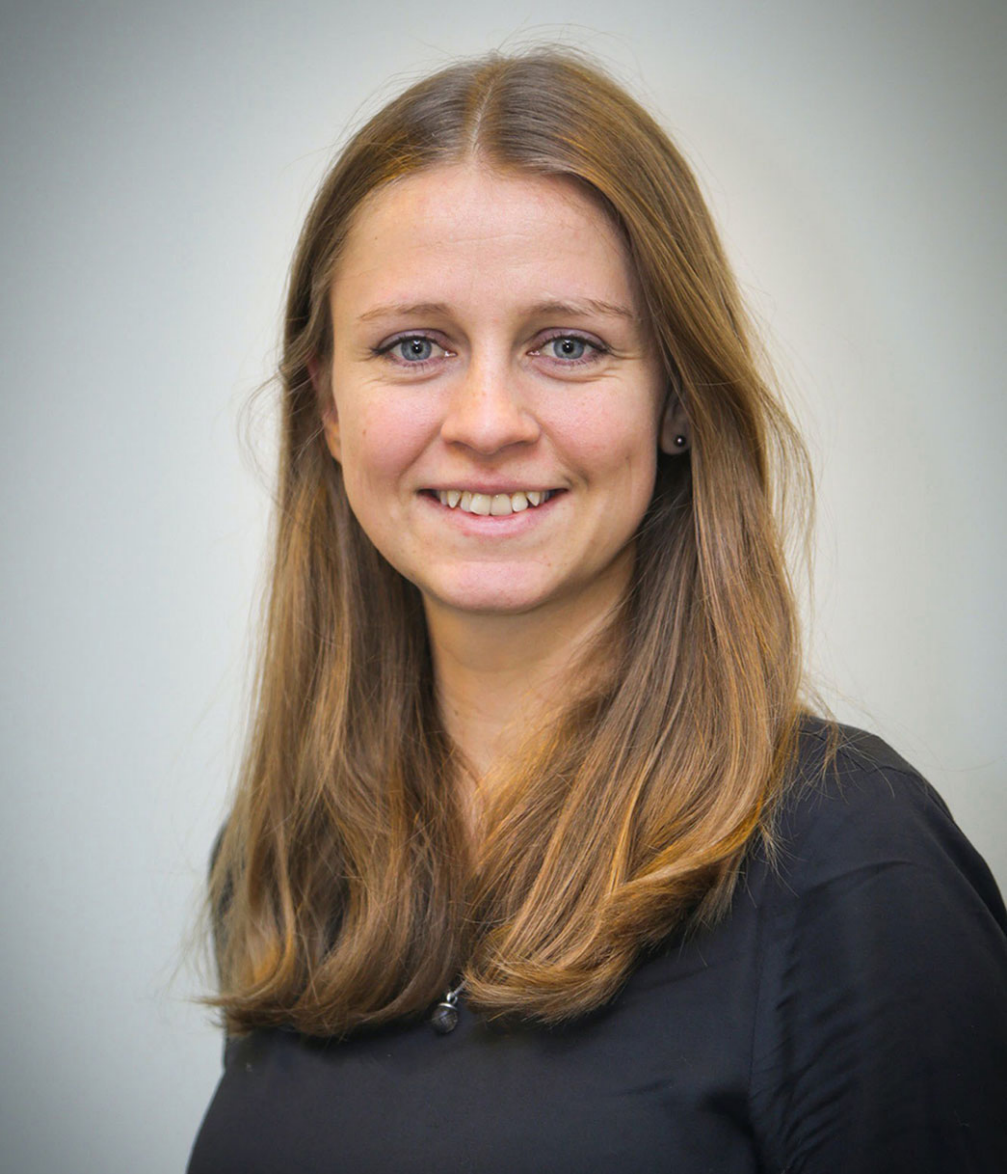


**Astrid M. Baattrup**

**Who or what inspired you to become a scientist?**

I cannot point to one specific thing or person that inspired me to become a scientist, but my high-school biology teacher, who was very engaging and passionate, played a big role in me deciding to study biomedicine and ultimately become a scientist.

**What is the main question or challenge in disease biology you are addressing in this paper? How did you go about investigating your question or challenge?**

Enteroendocrine cells in the gut are responsible for secreting a multitude of hormones that are crucial for regulating our metabolism; but, due to cell scarcity, they have historically been difficult to study. The emergence of organoid technology has provided a valuable model to study enteroendocrine cells, and we take advantage of this model in our work to study how expression of the hormone PYY is regulated.

**How would you explain the main findings of your paper to non-scientific family and friends?**

Specialised cells in the gut produce hormones that regulate various important functions throughout our body. One of these hormones is called PYY and is involved in regulating digestion and appetite. In our study, we describe a new mechanism for how levels of PYY are regulated. The function of a cell is determined by which genes are active, and this is controlled by transcription factors. We show that a transcription factor called ETV1 is important for regulating levels of PYY. Thus, when we remove ETV1, we see less PYY, and when we increase the levels of ETV1, we also see higher levels of PYY. This finding helps improve the understanding of how gut hormone levels are controlled, which could help developing new ways to treat various metabolic disorders in the future.

**What are the potential implications of these results for disease biology and the possible impact on patients?**

PYY has long been known for its role in various aspects of metabolism – in particular, for its function as an appetite-controlling hormone. In recent years, PYY has additionally been implicated in direct control of nutrient absorption, as well as having a potential antimicrobial function. Thus, improved understanding of how PYY expression is regulated could have therapeutic implications in treatment of various metabolic diseases, such as diabetes, obesity and malnutrition, and could also have therapeutic relevance in patients experiencing decreased appetite, e.g. due to chemotherapy.

… improved understanding of how PYY expression is regulated could have therapeutic implications in treatment of various metabolic diseases …

**Figure:**
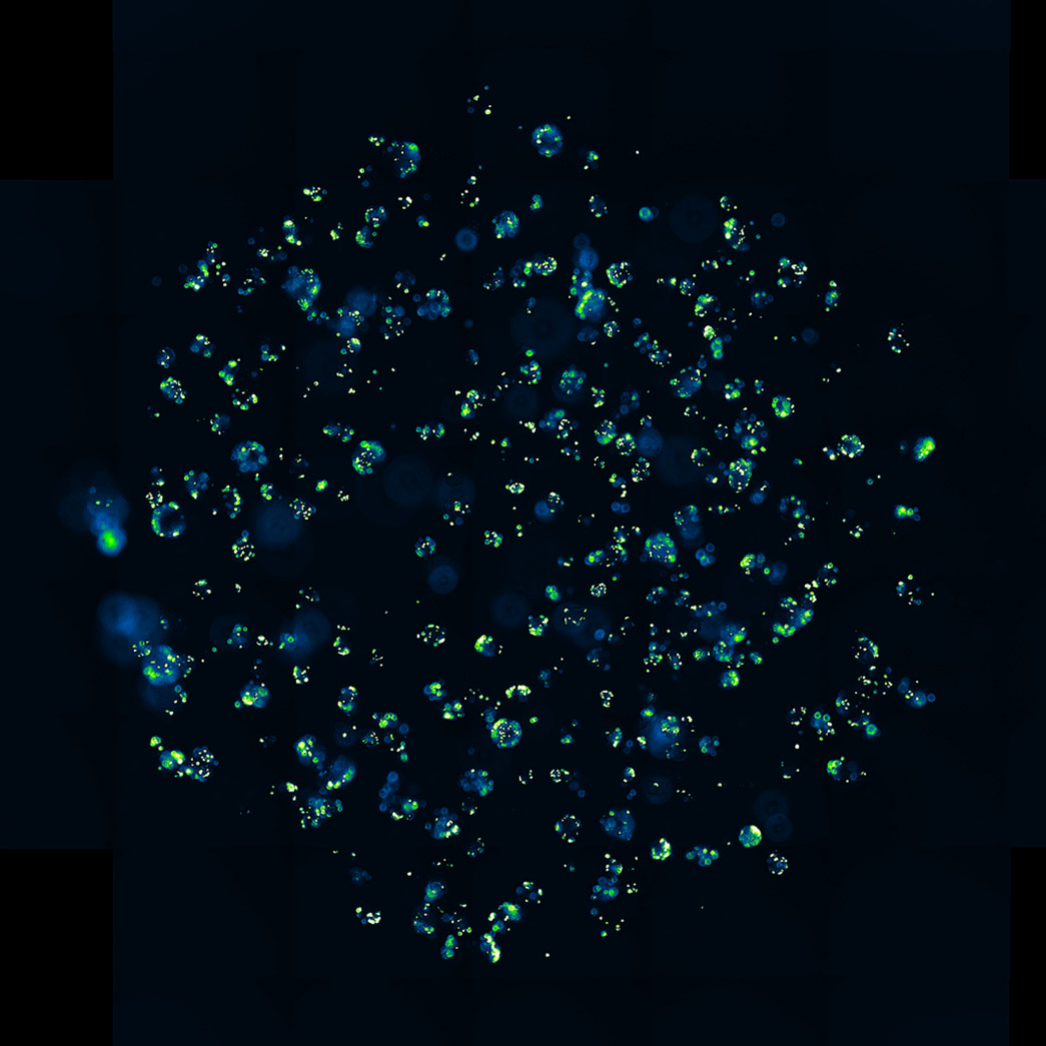
An overview of an intestinal organoid culture in which a subset of enteroendocrine cells is marked by a fluorescent marker.

**Why did you choose DMM for your paper?**

I chose to publish in DMM, because I appreciate that The Company of Biologists is a non-profit publishing organisation run by scientists, and our research aligns well with the scope of the journal.

**Given your current role, what challenges do you face and what changes could improve the professional lives of other scientists in this role?**

I think it could be beneficial to have more interaction between academia and industry. Ultimately, both are interested in furthering great science, which can form the basis of treating or curing diseases. The limited number of permanent research positions within academia also means that the majority of PhD and postdocs will end up in positions outside academia. Thus, for career development purposes, increased interaction between academia and industry could also facilitate building networks and highlight different career opportunities.

… for career development purposes, increased interaction between academia and industry could facilitate building networks and highlight different career opportunities

**What's next for you?**

I'm currently working on an exciting project in which I'm trying to set up a large compound screen using human organoids to screen for compounds affecting enteroendocrine hormone levels.

**Tell us something interesting about yourself that wouldn't be on your CV**

My favourite type of holiday is to go cross-country skiing.
